# Utility of Operative Glaucoma Tube Shunt Viscoelastic Bolus Flush

**DOI:** 10.5005/jp-journals-10008-1188

**Published:** 2016-02-02

**Authors:** Sylvia L Groth, Kelsi L Greider, William Eric Sponsel

**Affiliations:** Resident, Department of Ophthalmology, University of North Carolina Chapel Hill, North Carolina, USA; Resident Physician, Chicago Medical School, Chicago, Illinois, USA; Director and Professor, WESMD Professional Association, Baptist Medical Center Biomedical Engineering, University of Texas, Texas and Vision Sciences, University of the Incarnate Word, San Antonio, Texas USA

**Keywords:** Bleb fibrosis, Glaucoma surgery, Glaucoma tube shunt, Intraocular pressure, Modification/revision of glaucoma tube shunt, Ocular hypertension, Viscoelastic.

## Abstract

**Objective:** To assess the utility of viscoelastic injection to induce bleb expansion and decrease intraocular pressure (IOP) in eyes with encapsulated glaucoma tube shunt blebs.

**Design:** Case series.

**Subjects and participants:** Forty-three glaucomatous eyes, including 13 eyes with congenital, 13 uveitic, 5 neovascular, 5 open angle, 4 narrow angle and 3 traumatic glaucomas.

**Methods, interventions or testing:** All patients underwent viscoelastic flush procedure. A pre-bent 27 or 30-gauge cannula was passed through a 25-gauge paracentesis, advanced over the iris across the anterior chamber, and insinuated into the tube shunt lumen. Once the cannula was firmly lodged in position, 0.45 to 0.85 ml of viscoelastic was injected to hyperinflate the bleb.

**Main outcome measures:** Paired t-tests were performed comparing preoperative IOP and number of medications used preoperatively *vs* levels measured at 1, 6, 12, 18 and 24 months.

**Results:** Intraocular pressure was reduced from a mean preoperative level of 26.0 ± 1.2 (sem) mm Hg to 15.8 ± 1.0 at 1 month, remaining stable thereafter at each 6-month interval with 15.1 ± 1.1 mm Hg at 24 months (p < 0.0001). Medication use did not vary significantly from baseline. Pressure remained < 21 mm Hg after 2 years in 85% of eyes cannulated within 1 year of primary tube shunt implantation (n = 23), and in 62% of eyes cannulated more than 1 year after tube shunt placement (n = 20).

**Conclusion:** Tube shunt expansion with bolus viscoelastic flush successfully restored encapsulated bleb function, providing a substantial (~10 mm Hg) IOP decrease into the mid-normal pressure range. This persisted in the majority of treated eyes for the entire study period.

**How to cite this article:** Groth SL, Greider KL, Sponsel WE. Utility of Operative Glaucoma Tube Shunt Viscoelastic Bolus Flush. J Curr Glaucoma Pract 2015;9(3):73-76.

## INTRODUCTION

Chronic glaucoma can be managed both medically and surgically. When other measures fail to control intraocular pressure (IOP), tube shunts are commonly used to facilitate drainage of aqueous humor from the anterior chamber. Early complications that may occur after glaucoma tube shunt surgery include hypotony with or without hyphema, irideal obstruction, and uveitic, or conjunctival inflammatory reactions that may contribute to excessive capsular fibrosis.^1-3^ Late complications may include conjunctival erosion with implant or suture exposure, implant migration, valve occlusion, or chronic encapsulation with increase in IOP. A multicenter clinical trial observed a 29.8% failure rate of tube shunts after 5 years.^4^ This failure was overwhelmingly the consequence of fibrotic encapsulation of the bleb.^5,6^ Clinical maneuvers directed toward restoring function at the slit lamp include bleb massage combined with anterior chamber ballottement. Such maneuvers, if carried out in the early postoperative phase, can frequently successfully breach the lining of a newly encapsulated bleb, but such efforts are generally unsuccessful after the fibrin lining has become cross-linked. Transient resumption of medical therapy to manage the postoperative hypertensive phase that often arises 4 to 6 weeks after primary tube shunt placement is sometimes successful. However, a substantial proportion of blebs requiring such mitigation ultimately provide unsatisfactory pressure control. Failure of intractable encapsulation of tube shunts has been managed by internal debulking bleb revision procedures to attempt to restore pressure control to adequate levels. Debulking bleb revisions have been performed with YAG laser membranectomy,^7-9^ cyclophotocoagulation,^10,11^ 5-fluorouracil,^12^ tissue plasminogen activator,^13^ and by cannulation of tube with balanced salt solution flush.^14^ Tube shunt failure has also been managed by sequential additional tube placement.^15^ Performing additional incisional surgery in an eye already demonstrably prone to a fibrotic healing response may ultimately be the only option in certain instances, but less invasive surgical approaches to restoring the function of a failed tube shunt would be far preferable whenever possible. Prior research has shown that surgical revision of tube shunt implants provides limited long-term benefit when the failure is due to tube occlusion from fibrosis.^16^

Transcameral injection of viscoelastic into the reservoir via the tube can breach obstructed tubes, protein-sealed bileaflet valves, and mild to moderate bleb encapsulation with minimal inflammatory impact, potentially obviating the need for more aggressive surgery. This study analyzes the efficacy of rapid injection of viscoelastic via transcameral cannulation of the tube shunt orifice within the anterior chamber to clear the seton, inducing bleb expansion in an effort to decrease IOP.

## METHODS

This quality assurance analysis case series study was performed on all glaucomatous eyes undergoing a viscoelastic flush procedure to lower IOP over a 4-year period. Informed consent was obtained from each patient, and risks, benefits and alternatives to treatment were discussed. All information was stored in accordance with HIPAA, and IRB approval for this analysis was obtained through the Baptist Health Care System Institutional Review Board. The research was conducted in accordance with Declaration of Helsinki.

All procedures were performed in the operating room under the stereomicroscope. A 27 or 25-gauge paracentesis was created opposite the proximal tube in the anterior chamber and a straightened 30 or 27-gauge cannula was passed through the paracentesis, avoiding the lens, insinuated into the tube shunt lumen, and an entire vial (0.45 to 0.85 ml) of standard viscoelastic [Healon (Abbott Medical Optics, Abbott Park, IL), Viscoat (Alcon, Fort Worth, TX), Provisc (Alcon, Fort Worth, TX)] was injected to hyperinflate the bleb. The viscoelastic was left *in situ* in both the reservoir and anterior chamber without balanced salt solution rinse-out. The corneal entry site was then hydrated via cannula, and the eyes were treated with topical Maxitrol ointment (Alcon, Fort Worth, TX), a patch and fox shield, and subsequently received a 1 month tapered regimen of topical antibiotic [Vigamox (Alcon, Fort Worth, TX)] and topical prednisolone acetate [(Omnipred (Alcon, Fort Worth, TX)]. Paired t-test assessments of preoperative IOP and medications used were performed against baseline IOP values at 1, 6, 12, 18 and 24 months.

## RESULTS

Forty-three eyes of 43 patients underwent viscoelastic tube shunt flush procedures. Included were eyes of 13 patients with congenital, 13 uveitic, 5 neovascular, 5 open-angle, 4 narrow angle, and 3 with traumatic glaucoma. Of the 43 eyes that were treated, 23 eyes underwent the viscoelastic bleb re-expansion within 12 months of primary tube shunt implant surgery, and 20 eyes were cannulated > 12 months later. Mean preoperative IOP among the 43 eyes was 26.0 ± 1.2 (sem) mm Hg, improving to 15.8 ± 1.0 mm Hg at 1 month (p < 0.0001), sustained within the normal range at 16.4 ± 0.9 mm Hg, 170 ± 1.0, 14.4 ± 0.9 and 15.1 ± 1.1 mm Hg at 6, 12, 18 and 24 months, respectively. Preoperative meds were 0.84 ± 0.2 and remained relatively stable at 0.57 ± 0.2, 0.78 ± 0.2, 0.84 ± 0.2, 1.14 ± 0.2 and 0.93 ± 0.2 at 1, 6, 12, 18 and 24 months, respectively. Pressure remained < 21mm Hg at 24 months in 85% of eyes cannulated within 1 year of the primary implant procedure, and in 62% of eyes cannulated more than 1 year after the primary implant. At 2 years, 100% of those with angle closure or neovascular glaucoma, 75% with uveitic or open-angle glaucoma, 67% with congenital or traumatic glaucoma had pressures remaining ≤ 21 mm Hg. Table 1 displays the demographics of the 43 subjects in order of the magnitude of their mean level of postcannulation IOP reduction from baseline at the 12-month interval.

## DISCUSSION

Tube shunt placement offers reduction in IOP for many patients that fail medical, laser and surgical management of glaucoma. Unfortunately, tube shunt failure is a well-recognized sequel that may lead to a clinically unsatisfactory increase in IOP with time. The results of this study show that operative flushing of tube shunts is an effective technique that can be used to decrease IOP in patients that have previously undergone tube shunt placement when IOP remains unsatisfactory despite medical management. This straightforward surgical maneuver offers a valuable alternative to sequential tube shunt placement which requires more extensive surgical intervention and associated risks. We, therefore, recommend attempting transcameral flushing of tube shunts as a prospective solution to tube shunt failure before resorting to more invasive surgical options.

In the present study, patients with neovascular glaucoma, chronic uveitis and open angle glaucoma showed the most significant improvement for a sustained duration. There was a weak inverse association between the time elapsed between the initial implant procedure and performance of the viscoelastic flush; 85% of eyes cannulated within 12 months of the initial procedure maintained normal IOP for the ensuing 2 years, compared with 62% of those cannulated more than 1 year after their primary implant procedure.

**Table 1:** **Table 1:** Demographics of the 43 subjects showing intraocular pressures and number of ocular hypotensive medications in use immediately prior to and at the 12-month interval after undergoing viscoelastic tube shunt flush. Subjects are listed in order of the magnitude of intraocular pressure reduction attained

*Age*		*Gepder*		*Postshut*		*Gliucoma etiology*		*Eye*		*Preop IOP*		*12-month IOP*		*IOP change*		*Preop meds*		*12-month meds*	
54		M		1		Traumatic		L		56		9		–47		0		0	
47		M		4		Neovascular		R		40		16		–24		5		0	
81		F		8		Uveitic		R		38		15		–23		3		3	
41		F		16		Uveitic		R		28		7		–21		1		0	
41		F		2		Uveitic		R		34		13		–21		0		0	
71		F		8		Uveitic		R		33		14		–19		0		0	
50		F		3		Congenital		L		39		10		–19		0		0	
62		M		54		Neovascular		L		27		9		–18		0		0	
87		M		1		POAG		R		36		19		–17		0		0	
57		M		3		Neovascular		L		32		15		–17		0		0	
78		F		15		Uveitic		L		32		15		–17		0		0	
78		F		2		CACG		R		34		18		–16		0		0	
75		M		8		Uveitic		L		30		14		–16		0		0	
80		F		55		CACG		R		27		12		–15		1		0	
1		M		7		Congenital		R		24		12		–12		0		0	
49		F		1		POAG		L		34		22		–12		0		0	
75		M		8		Uveitic		L		25		14		–11		0		0	
14		M		9		Congenital		L		23		14		–9		1		0	
54		F		19		POAG		L		24		15		–9		0		0	
43		M		111		Congenital		L		21		12		–9		0		0	
62		F		154		Neovascular		R		24		15		–9		1		1	
1		M		1		Congenital		R		23		15		–8		0		0	
53		M		1		Uveitic		L		30		22		–8		0		0	
79		F		4		Uveitic		L		29		22		–7		0		0	
83		F		21		POAG		L		28		21		–7		3		2	
22		F		3		Congenital		L		26		20		–6		2		2	
10		F		11		Congenital		L		24		18		–6		1		1	
4		M		60		Traumatic		L		19		15		–4		0		0	
56		F		31		POAG		L		19		16		–3		0		0	
76		M		5		NAG		R		16		13		–3		0		0	
4		M		21		Congenital		R		23		20		–3		0		0	
72		F		4		Uveitic		R		21		18		–3		0		0	
5		M		42		Congenital		R		18		16		–2		2		2	
6		M		38		Congenital		L		19		17		–2		2		0	
42		F		2		Neovascular		L		28		26		–2		0		0	
9		M		95		Congenital		L		23		21		–2		3		4	
77		F		136		ACG		L		16		15		–1		0		0	
7		M		7		Congenital		R		20		22		2		0		0	
54		F		51		Uveitic		L		19		21		2		1		2	
76		M		9		Uveitic		L		28		33		5		0		0	
87		F		38		Uveitic		L		28		33		5		0		0	
3		M		31		Congenital		R		25		33		8		0		0	
97		R		2		Traumatic		L		21		29		8		3		3	

M: Male; F: Female

Bleb encapsulation will likely continue to be a consistent challenge for clinicians and glaucoma patients. The more effective tools made available to combat this perennial source of intransigent ocular hypertension, the more successful the management of difficult glaucoma cases will become. Innovations directed toward overcoming bleb encapsulation that does not respond to conservative measures like massage, medication, or cannulation remain a priority. Among our patient population, individual s who failed to respond adequately to tube shunt flush became candidates for a new posterior extension implant that effectively converted the impervious bleb as an intermediate cistern en route to the retrobulbar fat pad, where aqueous reuptake was efficient and sustained.^17,18^ This approach and others may ultimately help facilitate long-term pressure control in patients with intractable ocular hypertension after tube shunt surgery, but we believe the less invasive tube shunt flush maneuver should always be attempted before placing another surgical implant.
